# PTH 1-34 promoted bone formation by regulating iron metabolism in unloading-induced bone loss

**DOI:** 10.3389/fendo.2022.1048818

**Published:** 2023-02-02

**Authors:** Jingmin Che, Weihao Ren, Xin Chen, Fang Wang, Gejing Zhang, Peng Shang

**Affiliations:** ^1^ Research & Development Institute of Northwestern Polytechnical University in Shenzhen, Shenzhen, Guangdong, China; ^2^ Shaanxi Provincial Key Laboratory of Infection and Immune Diseases, Shaanxi Provincial People’s Hospital, Xi’an, China; ^3^ School of Life Sciences, Northwestern Polytechnical University, Xi’an, Shaanxi, China; ^4^ Key Laboratory for Space Bioscience and Biotechnology, Northwestern Polytechnical University, Xi’an, Shaanxi, China

**Keywords:** unloading, PTH 1-34, bone formation, iron metabolism, NRF2

## Abstract

PTH 1-34 (teriparatide) is approved by FDA for the treatment of postmenopausal osteoporosis. Iron overload is a major contributing factor for bone loss induced by unloading. Whether iron metabolism is involved in the regulation of PTH 1-34 on unloading-induced osteoporosis has not yet been reported. Here, we found that PTH 1-34 attenuated bone loss in unloading mice. PTH 1-34 regulated the disturbance of iron metabolism in unloading mice by activating Nrf2 and further promoting hepcidin expression in the liver. In addition, the Nrf2 inhibitor selectively blocked hepcidin expression in the liver of unloading mice, which neutralized the inhibitory effect of PTH 1-34 on bone loss and the recovery of iron metabolism in unloading mice. Finally, we found that PTH 1-34 promoted the differentiation and inhibited apoptosis of osteoblasts by regulating iron metabolism and maintaining redox balance under unloading conditions. Our results suggested that PTH 1-34 promoted bone formation by regulating iron metabolism under unloading conditions.

## Introduction

Long-duration spaceflight induces bone loss in astronauts, especially weight-bearing bones (1). The loss rate of the lower limb in astronauts on term missions is 0.5% to 1% per month, which is approximately 10-fold greater than those observed in postmenopausal women (2, 3). For ground-based modeling, hindlimb unloading (HLU) is a widely used rodent model that has the mechanical loading and fluid flow similar to astronauts. After two to three weeks of HLU treatment, the weight-bearing bones of mature mice exhibit bone loss similar to that seen in astronauts (4, 5). Accumulating evidence showed that iron overload is a key factor to induce bone loss in microgravity (6). Astronauts exhibited an increase in iron storage and serum ferritin after short- and long-duration spaceflight (7, 8). Higher iron content is associated with higher levels of oxidative damage, and with a greater degree of reductions in bone mineral density (BMD) in astronauts after long space-flight (9). Recently studies showed that bone loss induced by HLU is associated with systemic iron overload, which is due to disturbed hepcidin expression in the liver (10–12).

Previous studies have reported that the inhibition of bone formation is the main cause of HLU-induced bone loss (13). Parathyroid hormone (PTH) is a calciotrophic hormone produced by parathyroid glands and a critical regulator of skeletal development (14). PTH 1-34 (teriparatide) is a biologically active N-terminal fragment of human PTH (15). PTH specifically interacts with the parathyroid hormone type-1 receptor (PTH1R) which is a member of the G-protein-coupled receptor (GPCR) family and highly expressed in bone cells, liver cells and kidney cells (16, 17). Once-daily administration of PTH increases bone mass (bone anabolism) by promoting osteogenic differentiation of bone marrow mesenchyme stem cells (BMSCs), preventing mature osteoblast apoptosis, and activating lining cells (18, 19), while continuous administration induces bone loss (bone catabolic) (20). Teriparatide (PTH 1-34) is the most widely prescribed bone anabolic drug in the world (21). However, the feasibility of its application in microgravity-induced bone loss is highly controversial. Studies have reported that once-daily administration of PTH could be clinically effective for unloading-induced bone loss (22–25). Other studies showed that the bone anabolism of PTH was attenuated in unloading-induced bone loss (26) and actions of PTH on cancellous bone were independent of the level of mechanical usage (27). Furthermore, the molecular mechanisms involved are not clearly understood.

Excess iron catalyzes the formation of pro-oxidants, affects the redox balance and causes tissue damage (28). PTH-related protein inhibits the generation of ROS in osteoblasts by regulating and activating the phosphorylation of MKP1 (29). PTH 1-34 can reduce the cellular ROS level caused by glucocorticoids and facilitate the proliferation of osteocytes by activating the AKT pathway (30). Evidence from 42 patients with idiopathic calcium pyrophosphate crystal deposition showed that the serum ferritin content was inversely proportional to PTH (31). Whereas, whether iron metabolism is involved in PTH-regulated bone formation in HLU mice has not been reported. In the present study, we used the HLU model and random positioning machine (RPM) to stimulate weightlessness and tried to investigate the effect of PTH 1-34 on HLU-induced bone loss in male C57BL/6 mice and elucidate the possible molecular mechanism of PTH 1-34 on HLU-induced bone loss *via* iron metabolism. This study not only provides a new molecular mechanism underlying PTH 1-34 induced bone formation but also offers new ideas for possible drug targets for osteoporosis caused by iron overload.

## Materials and methods

### Animals and treatment

**Figure 1:**
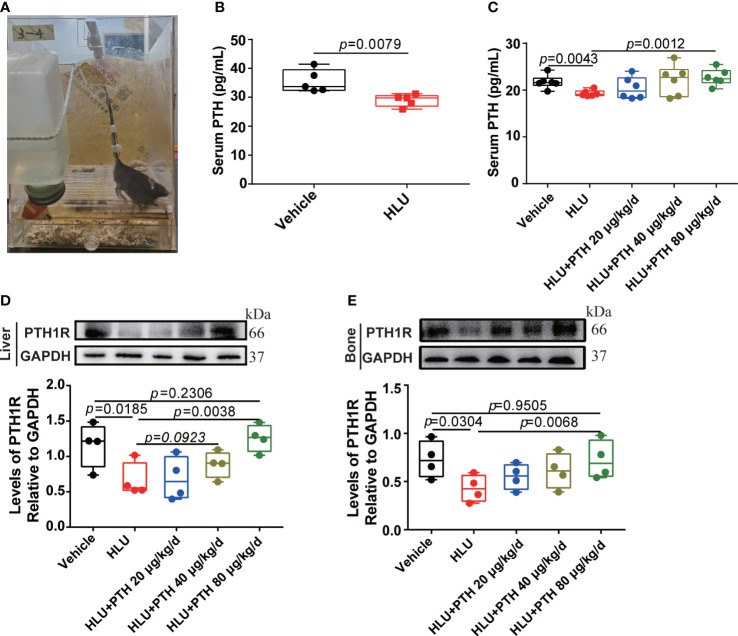
PTH 1-34 stimulated PTH1R expression in the liver and bone of HLU mice. **(A)** the HLU model. **(B)** and **(C)** The concentration of PTH in serum was determined by mouse PTH ELISA kit (n = 5-6). **(D)** The expression and quantitative analysis of PTH1R in liver. **(E)** The expression and quantitative analysis of PTH1R in bone. (n = 4). Data is shown as the mean ± SEM.

Male C57BL/6 mice were purchased from the Experimental Animal Center of Xi’an Jiaotong University (6-7 weeks old, 18 ± 2g). All mice were housed at an ambient temperature of 24 ± 2°C under an artificial 12 h light/dark cycle with food and water provided, at least one week before carrying out experiments. These mice were randomly divided into five groups (Vehicle, HLU, HLU+PTH 20 μg/kg/d, HLU+PTH 40 μg/kg/d, HLU+PTH 80 μg/kg/d) and subcutaneously injected with an equal volume of saline with or without different concentration of PTH 1-34 (Sangon Biotech, China) for 28 d. In the reverse validation scheme, 40 male C57BL/6 mice were divided into Vehicle, HLU, HLU+PTH, HLU+ML385 (T4360, TargetMol, China), HLU+ML385+PTH, where the dose of PTH 1-34 was 80 μg/kg/d, and the dose of ML385 was 30m/kg/7d. The HLU model was established by using the NASA-designed tail suspension system which was modified by Morey-Holton to simulate weightlessness (32) (**Figure 1A**). Animal protocols were approved by the Ethics Committee of Northwestern Polytechnical University.

### BMC and BMD evaluation

Bone mineral content (BMC) and bone mineral density (BMD) of the femur were analyzed by using Dual-energy X-ray absorptiometry (DEXA) (InAlyzer; MEDIKORS, Korea). In brief, after the experiment, all mice were anesthetized and placed in the absorptiometry machine for BMD and BMC measurement. The scanning results were analyzed with dual X-ray digital imaging software (InAlyzer; MEDIKORS, Korea) to assess the BMD and BMC of whole femurs.

### Micro-computed tomography imaging

The femur was scanned by *μ*CT scanner (SkyScan 1176; Bruker, Kontich, Belgium). Scanning was performed at 80 kV, 305 mA, 525 ms without filter. In total, 421 projections were collected per sample at a resolution of 8.96 μm per pixel. Then, these scan results were reconstructed by NRecon software and analyzed by CTAn software. The area-of-interest (ROI) was selected by employing an automated algorithm. 1-mm-long ROI were selected for analysis of trabecular bone (distance from the distal growth plate 0.5 mm to 1.5 mm) and the grayscale value was set at a threshold of 76. Other 1-mm-long ROI were selected for analysis of cortical bone (distance from the distal growth plate 5 mm to 6 mm) and the grayscale value was set at a threshold of 105. All trabecular bone, from each selected slice, was segmented for three-dimensional reconstruction to calculate the following parameters: bone volume/total bone volume (BV/TV, %), trabecular separation (Tb.Sp, mm) and trabecular number (Tb.N, 1/mm). All cortical bone, from each selected slice, was segmented for three-dimensional reconstruction to calculate the following parameters: tickness (Tb.Th, mm), cortical mean thickness (Ct.Th, mm), Ct.Ar (cortical area, mm^2^) and total cross-sectional tissue area (Tt. Ar, mm^2^) (33).

### Biomechanical testing

The load-displacement curve was obtained from software (Instron 5943, Instron, Canton, MA). Displacement and force were used to calculate parameters that describe the whole bone’s structural properties. The mechanical characteristics of the femur and analytical methods refer to those previously reported (34). The mechanical characteristics parameters of the femur include stiffness (N/mm), Ultimate Load (N), Elastic Modulus (GPa) and Ultimate stress (MPa).

### Biochemical assay

The blood samples were centrifuged and the serum was collected. The PTH, hepcidin, ALP and OCN in serum were measured by using mouse enzyme-linked immunosorbent assay (ELISA) kits. Mouse PTH ELISA kit (JYM0697Mo, Jiyinmei Biotech, China), mouse hepcidin ELISA kit ((JL20572, Jianglai Biotech, China), ALP ELISA kit (JL26471, Jianglai Biotech, China) and mouse OCN ELISA kit (JYM0432Mo, Jiyinmei Biotech, China) were used in these assays, and all steps followed the manufacturer’s instructions.

### Histological analysis

Liver, spleen and right femur of mice were fixed in 4% paraformaldehyde for 24 h. The femur was decalcified treatment by using 10% EDTA for 24 d. Then, femur, liver and spleen were embedded in paraffin and sectioned into 5 μm-thick slices. The femur sections were stained with hematoxylin and eosin (H&E; C0105S, Beyotime, China) for analyzing the osteoblast number of the bone surface and with TRAP kit (Sigma-Aldrich, USA) for analyzing osteoclast number of bone surface, respectively. These liver and spleen sections were stained with Prussian blue (234125, Sigma-Aldrich, USA), then developed with DAB (D3939, Sigma-Aldrich, USA) and counterstained with hematoxylin for the iron deposition analysis.

### Cell culture and experimental design

MC3T3-E1 osteoblasts were provided by Prof. Hong Zhou from the University of Sydney. Primary osteoblasts were derived from the cranium of C57BL/6 (3-5 d old, male). Cells grown in α-Minimum Essential Medium (α-MEM; Gibco) supplemented with 10% (v/v) fetal bovine serum (FBS; Gibco) and 100 units/mL penicillin-streptomycin at 37°C in a 5% CO_2_ incubator. For differentiation and mineralization experiments, the growth medium was supplemented with fresh ascorbic acid (50 μg/mL, A4544, Sigma-Aldrich, USA) and β-glycerolphoate (5 mM, G9422, Sigma-Aldrich, USA).

Cells were exposed to different conditions: (1) Control: cells were exposed to control 1g force conditions; (2) Random Positioning Machine (RPM, stimulate disuse or microgravity conditions): cells were continuously rotated on the RPM, stimulating a microgravity environment; (3) PTH: cells were exposed to control 1g force conditions with PTH 1-34 (100 nM); (4) RPM-PTH: cells were continuously rotated on the RPM with PTH 1-34 (100 nM); (5) RPM-FAC: cells were continuously rotated on the RPM with FAC (50 μM, Sigma-Aldrich, USA); (6) RPM-FAC-PTH: cells were continuously rotated on the RPM with FAC (50 μM) and PTH 1-34 (100 nM); (7) RPM-DFO: cells were continuously rotated on the RPM with DFO (10 μM, Sigma-Aldrich, USA); (8) RPM-DFO-PTH: cells were continuously rotated on the RPM with DFO (10 μM) and PTH 1-34 (100 nM); (9) RPM-NAC: cells were continuously rotated on the RPM with NAC (1 mM, Sigma-Aldrich, USA); (10) RPM-NAC-PTH: cells were continuously rotated on the RPM with NAC (1 mM) and PTH 1-34 (100 nM).

### Cell apoptosis assay

Apoptosis was detected by an Annexin V-FITC/PI apoptosis detection kit (556547, BD Biosciences, San Jose, CA, USA) and expression levels of apoptosis-related proteins. MC3T3-E1 cells were seeded in a 35 mm plate at a density of 7.5×10^4^ cells/mL. Cells were harvested after RPM with or without 100 nM PTH 1-34 treatment for 48 h and washed with PBS. Then, the cells were stained with Annexin V- FITC and PI for 20 min in the dark and analyzed by an MD IL HC inverted fluorescence microscope (Leica, Wetzlar, Germany) and the quantification of apoptosis cell was analyzed by Image J software. For expression levels of apoptosis-related proteins (Bcl-2, Bax and cleaved caspase 3), cells were washed with pre-cold PBS and lysed by RIPA. The supernatant was collected centrifugally, then the expression levels of proteins were analyzed by western blot.

### RNA isolation and RNA-seq analysis

MC3T3-E1 cells were seeded in a 35 mm plate at a density of 7.5×10^4^ cells/mL. Cells were harvested after RPM with or without 100 nM PTH 1-34 treatment for 48 h. Total RNA was extracted from treated cells using TRizol reagent (15596018, ThermoFish, USA). After quality control, the cDNA libraries were prepared, and sequencing was performed by Novogene Technology Co. Ltd. (Tianjin, China). Raw reads were generated and filtered. The clean reads were used for mapping to the iron metabolism reference genome using Hisat2 v2.0.5 software. Gene expression was determined using featureCounts (v1.5.0-p3) and normal using Fragments Per Kilobase of transcript sequence per Millions. The differential expression among different groups was identified by the DESeq2 package. Genes with an adjusted *P*-value < 0.05 found by DESeq2 were assigned as differentially expressed. And the differential expression genes used online resources (https://david.ncifcrf.gov) were implemented in DAVID to conduct the gene ontology overrepresentation analysis and pathway analysis.

### Iron metabolism evaluation experiment

The iron metabolism was evaluated by analyzing the intracellular total iron, ferrous ion content and the expression of iron metabolism-related proteins.

For total iron content, the attached cells were treated with given different conditions for 48 h or 8 d. Cells were collected and washed with PBS. The tissue weight was determined, and the tissue were dried at 160 °C for 3 h and the dry weight of them measure. The dried tissue samples were ash in a resistance furnace (TAISITE, Tianjin, China) at 550 °C. These samples of tissue or cells for iron quantification were lysed with 65% HNO_3_ at 70°C for 2 h (11). Finally, the total iron level was determined by atomic absorption spectrometer (AAS) (Analytik, Jena, Germany). The cell iron content was normalized by protein concentration, and the tissue iron content was normalized by dry tissue weight (11).

For ferrous ion content, the attached cells were treated with given different conditions for 48 h or 8 d. Cells were collected and washed with PBS and analyzed by an iron assay kit (ab83366, Abcam, USA). The intercellular ferrous ion content was normalized by protein concentration.

### Mitochondrial function evaluation experiment

The mitochondrial function was assessed by analyzing mitochondrial membrane potential (MMP), mitochondrial morphology, intracellular ATP content and intracellular NAD+/NADH ratio. MC3T3-E1 cells were seeded in 35 mm plate at a density of 7.5×10^4^ cells/mL, and collected after 100 nM PTH 1-34 treatment with or without FAC (50 μM) or DFO (10 μM) pretreatment under RPM conditions for 24 h (MMP and mitochondrial morphology) or 48 h (ATP content and NAD+/NADH ratio).

Cells were digested with trypsin and collected, and washed with PBS. The MMP was measured with a JC-1 fluorescent probe (C2003S, Beyotime, China) and analyzed by a FACS Calibur flow cytometer (BD Biosciences, San Jose, CA, USA). For mitochondrial morphology, cells were washed with PBS and stained with 20 nM Mito-Tracker TM Green (M7514, ThermoFisher, USA) and imaged with a confocal microscope (Fluoview FV1000, Olympus, Tokyo, Japan). For intracellular ATP content, cells were washed with PBS and lysed with ATP detection lysate. The intracellular ATP content was analyzed by using ATP assay kit (C0026, Beyotime, China). For intracellular NAD+/NADH ratio, cells were lysed with NAD+/NADH extract and the NAD+/NADH ratio was analyzed by using NAD+/NADH assay kit with WST-8 (S0175, Beyotime, China).

### REDOX equilibrium evaluation experiment

The REDOX equilibrium experiments included the evaluation of intracellular reactive oxygen species (ROS), malondialdehyde (MDA) content, superoxide dismutase (SOD) content and reduced glutathione (GSH) content. MC3T3-E1 cells were seeded in 35 mm plate at a density of 7.5×10^4^ cells/mL and collected after 100 nM PTH 1-34 treatment with or without FAC (50 μM), DFO (10 μM) or NAC (1 mM) pretreatment under RPM conditions for 48 h.

For the intracellular ROS, cells were digested with trypsin and collected, and washed with PBS. The ROS content was measured by using the ROS assay kit (S0033, Beyotime, China) and analyzed by a FACS Calibur flow cytometer (BD Biosciences, San Jose, CA, USA). For MDA content and SOD content, cells were lysed with RIPA and the supernatant was collected. The MDA content was measured by using lipid peroxidation MDA assay kit (S0131S, Beyotime, China) and normalized by protein concentration. The SOD content was measured by using total superoxide dismutase assay kit with WST-8 (S0101S, Beyotime, China) and normalized by protein concentration. For GSH content, cells were washed PBS, and the GSH content was measured by using GSH assay kit (A006-2-1, Nanjingjiancheng Biotech, China) and normalized by protein concentration.

### Osteoblast differentiation assay

ALP activity was measured using alkaine phosphatase assay kit (P0321S, Beyotime, China), In brief, MC3T3-E1 cells of the four different groups (Control, RPM, PTH and RPM+PTH) in the differentiation period were induced by ascorbic acid (50 μg/mL) and β-Sodium 3-phosphoglycerate (5 mM) for 8 d and washed twice with PBS. Each sample was fixed with 4% paraformaldehyde for 30 min, then stained with BCIP/NBT alkaline phosphatase color development kit (C3206, Beyotime, China) for 2 h.

The mineralized nodule was analyzed using a 0.5% Alizarin Red S (A5533, Sigma, USA) solution. In brief, primary osteoblast cells of the four different groups (Control, RPM, PTH and RPM+PTH) in the differentiation period were induced by ascorbic acid (50 μg/mL) and β-Sodium 3-phosphoglycerate (5 mM) for 21 d. The cells were washed twice with ice-cold PBS and fixed with 70% ethanol for 15 min and stained for 15 min with a 0.5% Alizarin Red S solution for calcium detection.

### Western blot assay

After treatment, cells or tissue were washed with pre-cooled PBS and lysed with RIPA containing a protease inhibitor and phosphatase inhibitor. The protein concentration was determined using a bicinchoninic acid (BCA) protein assay kit according to the manufacturer’s instructions. Proteins of equal quality were separated by SDS-PAGE and then transferred onto a PVDF membrane. The membrane was sealed with 5% skimmed milk powder at room temperature for 2 h, then the primary antibodies against Bcl-2 (1:2000, ab182858, Abcam, USA), Bax (1:2000, ab182733, Abcam, USA), cleaved caspase 3 (1:1000, 9661T, Cell Signaling Technology, USA), ferritin (FTH1, 1:1000, 3998S, Cell Signaling Technology, USA), Ferroportin (FPN, 1:1000, ab78066, Abcam, USA), Transferrin receptor 1 (TfR1, 1:1000, 13113, Cell Signaling Technology, USA), hepcidin (1:1000, ab30760, Abcam, USA), Divalent metal ion transporter 1 (DMT1, 1:1000, 15083, Cell Signaling Technology, USA), NRF2 (1:1000, ab62352, Abcam, USA), bone morphogenetic protein 6 (BMP6, 1:1000, ab155963, Abcam, USA), GAPDH (1:10000, ab8245, Abcam, USA) were bound to the target protein at 4°C overnight. After washing with tris buffered saline with Tween-20 (TBST) solution, the membranes were incubated with horseradish peroxidase (HRP)-conjugated secondary antibody at room temperature for 2 h. Each immunoreactive band was detected by ECL on a T5200 multi automatic fluorescence/chemiluminescence imaging system (BioTanon, Shanghai, China) and quantified using ImageJ software.

### Statistics

Graphs and data statistical analyses were performed using GraphPad Prism software. Student’s *t*-test or one-way ANOVA were used to analyze the significant differences in groups. Data are presented as the mean ± standard error of the mean (SEM). In all cases, *P* < 0.05 was considered statistically significant.

## Results

### PTH 1-34 prevented the deterioration of bone microstructure and mechanical properties under unloading conditions

HLU mice model (**Figure 1A**) is a widely used rodent model that has mechanical loading and fluid flow similar to astronauts. Serum PTH levels decreased by 48% during astronaut flight (35). We found that serum PTH was significantly reduced in HLU mice, which was similar to that of astronauts (**Figure 1B**). Next, HLU mice were used to investigate the effects of PTH 1-34 on promoting bone formation. Firstly, the result of the serum PTH content showed that serum PTH content was decreased in HLU group relative to the vehicle group, while that was increased in HLU+PTH groups, especially in the HLU+ PTH 1-34 80 μg/kg/d group (**Figure 1C**). Next, we analyzed the PTH1R expression in HLU mice after PTH 1-34 injection daily for 28 d. We found that the expression of PTH1R in liver and bone was increased in HLU+PTH 1-34 40 μg/kg/d and 80 μg/kg/d groups, especially in the HLU+PTH 1-34 80 μg/kg/d. These results showed that serum PTH content and PTH1R expression were decreased in HLU mice, and PTH 1-34 inhibited the decreased serum PTH at 80 μg/kg/d and the decreased PTH1R expression in liver and bone at 80 μg/kg/d (**Figures 1D, E**).

**Figure 2:**
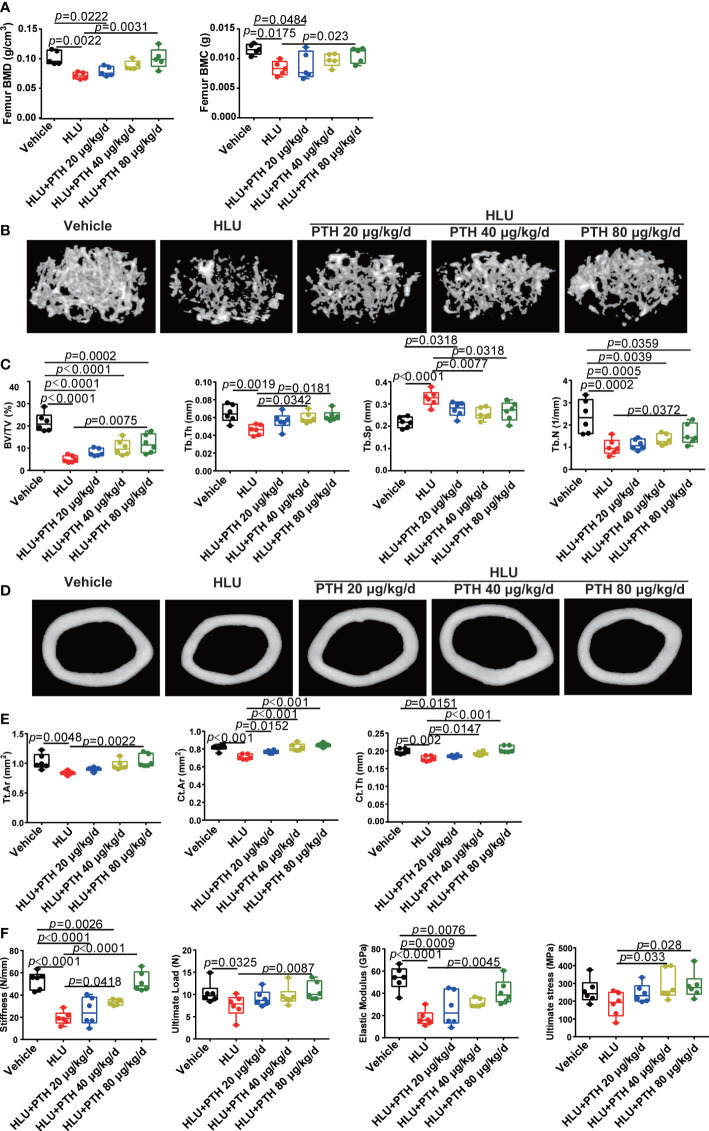
PTH 1-34 attenuated the deterioration of bone microstructure and mechanical properties in HLU mice. **(A)** BMC and BMD were measured by DEXA. **(B)** Three-dimensional images of trabecular architecture in the distal femur. **(C)** Structural parameters of trabecular bone. **(D)** Three-dimensional images of cortical architecture in the midshaft femur. **(E)** Structural parameters of cortical. **(F)** Mechanical properties of the tibia in mice were detected through three-point bending. (n =5 - 6). Data is shown as the mean ± SEM.

BMD and BMC are important markers of bone quality, reflecting the degree of osteoporosis (36). These results of BMD and BMC showed that the femoral BMD and BMC of the HLU groups were significantly decreased compared with the vehicle group, whereas PTH 1-34, especially 80 μg/kg/d, could increase femoral BMD and BMC under HLU stimulation (**Figure 2A**). The evaluation of bone tissue properties is an important means to understand bone degenerative diseases represented by osteoporosis. The analysis of bone structural morphology, bone trabecular structure and other factors could further improve and perfect the assessment of osteoporosis based solely on bone density (37). These results of some trabecular parameters of the proximal femur (BV/TV, Tb.Th, Tb.Sp and Tb.N) showed that these trabecular parameters of proximal femur significantly decreased in the HLU group compared with the vehicle group except Tb.Sp, while those were improved to near normal in the HLU mice treated with PTH 1-34, especially the Tb.Th and Tb.Sp in HLU+PTH 40 μg/kg/d and 80 μg/kg/d groups (**Figures 2B, C**). These results of bone micro-structure parameters of the tibia showed that parameters including Tt.Ar, Ct.Ar and Ct.Th of HLU mice were significantly decreased compared with that of mice in the vehicle group, while those of HLU + PTH 1-34 groups were improved, especially in the HLU+ PTH 1-34 40 μg/kg/d and 80 μg/kg/d groups (**Figures 2D, E**).

Bone is an organ that is sensitive to mechanical stimuli, which could help the bones grow correctly and keep strong, whereas the removal of mechanical stimuli could accelerate bone loss (38). These results of the mechanical properties of femur (stiffness, ultimate load,elastic modulus, and ultimate stress) showed that the stiffness, ultimate load and elastic modulus of the femur in the HLU group were significantly reduced than that in the vehicle group, while subcutaneous injection of PTH 1-34 could restore these changes under HLU stimulation, especially in the group with the administration of PTH 1-34 at 80 μg/kg/d. The ultimate stress of the femur in the HLU group was also light lower than the vehicle group, while that was promoted in the group of HLU with PTH 1-34 treatment (40 μg/kg/d and 80μg/kg/d) compared with the HLU group (**Figure 2F**). In summary, these results suggested that PTH 1-34 effectively prevents the deterioration of bone microstructure and mechanical properties in HLU mice

### PTH 1-34 promoted bone formation by maintaining the viability and differentiation of osteoblast under unloading conditions.

**Figure 3:**
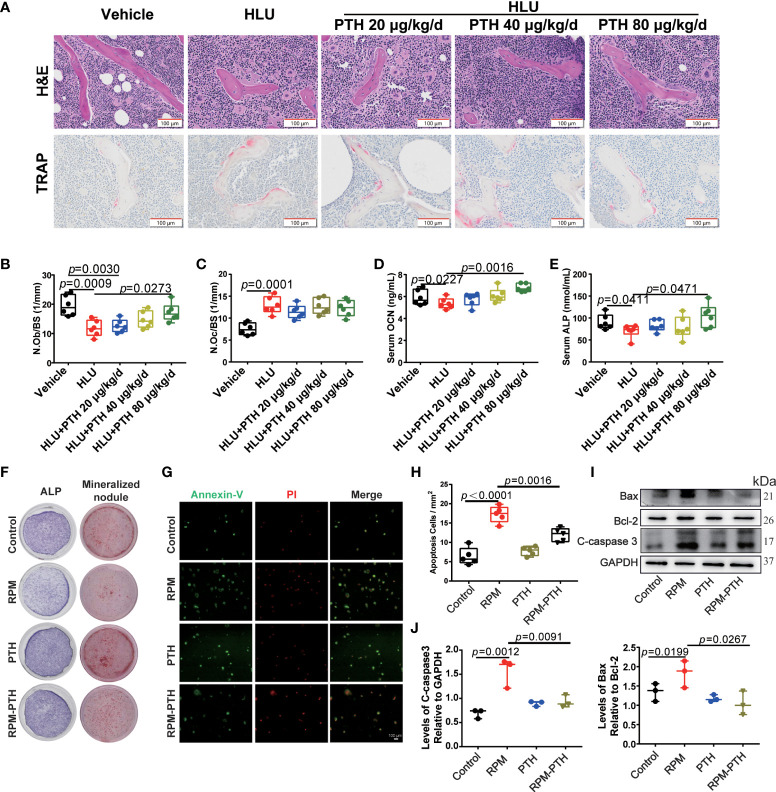
PTH 1-34 maintains bone formation by inhibiting apoptosis and promoting the differentiation of osteoblasts under unloading conditions. **(A)** H&E staining and TRAP staining of the trabecular bone distal to the femur in HLU mice (scale bar = 100 μm). **(B)** The number of osteoblasts on the trabecular surface was estimated by N.Ob/BS, (n = 6). **(C)** The number of osteoclasts on trabecular surface was estimated by N.Oc/BS, (n = 6). **(D, E)** Serum bone metabolism markers (OCN and ALP) were analyzed by ELISA kit. (n = 6). **(F)** The ALP stain and mineralized nodule of MC3T3-E1 cells treated with RPM conditions with or without 100 nM PTH 1-34. **(G)** Apoptosis analysis of MC3T3-E1 osteoblasts treated with RPM conditions with or without 100 nM PTH 1-34 by Annexin V-FITC/PI. **(H)** The quantification of apoptosis cells in MC3T3-E1 osteoblasts treated with RPM conditions with or without 100 nM PTH 1-34, (n = 5). **(I, J)** The expression levels and quantification analysis of apoptosis-related proteins in MC3T3-E1 osteoblasts treated with RPM conditions with or without 100 nM PTH 1-34. (n = 3). Data is shown as the mean ± SEM.

The disturbed bone remodeling is caused by the imbalance between bone formation regulated by osteoblasts and bone resorption mediated by osteoclasts. The results of the number of osteoblasts on the surface of the trabecular bone (N. Ob/BS) and the number of osteoclasts on the surface of the trabecular bone (N. Oc/BS) showed that N. Ob/BS was significantly decreased and N.Oc/BS was significantly increased in the HLU group compared with that of the vehicle group, and N.Ob/BS was rescued by PTH 1-34 in HLU mice, especially 80 μg/kg/d (**Figures 3A, B**), while the N.Oc/BS did not change significantly compared with that of the HLU group (**Figures 3A, C**). Based on the above results, we focus on the effect of PTH 1-34 on bone formation. alkaline phosphatase (ALP) and osteocalcin (OCN) are the key indexes for evaluating bone formation. These results showed that serum OCN content (**Figure 3D**) and serum ALP activity (**Figure 3E**) were significantly decreased in the HLU group compared with that of the vehicle group, which were rescued by PTH 1-34, especially PTH 1-34 80 μg/kg/d. To further verify the effect of PTH 1-34 on bone formation, we determined the differentiation and viability of osteoblasts which were cultured under random positioning machine (RPM, stimulate disuse or microgravity conditions) conditions with or without 100 nM PTH 1-34. These results showed that PTH 1-34 enhanced the expression levels of ALP in MC3T3-E1 cells and the formation of mineralized nodules in primary osteoblasts under RPM conditions (**Figure 3F**). Moreover, PTH 1-34 inhibited the apoptosis of osteoblasts (**Figures 3G, H**) and suppressed the expression of apoptosis-related proteins (Bax and C-caspase3) under RPM conditions (**Figures 3I, J**). These above results indicated that PTH 1-34 effectively inhibited apoptosis of osteoblasts, promoted osteoblast differentiation, and maintained bone formation under unloading conditions.

### Iron metabolism may be involved in the process of PTH 1-34 promoting bone formation under unloading conditions

**Figure 4:**
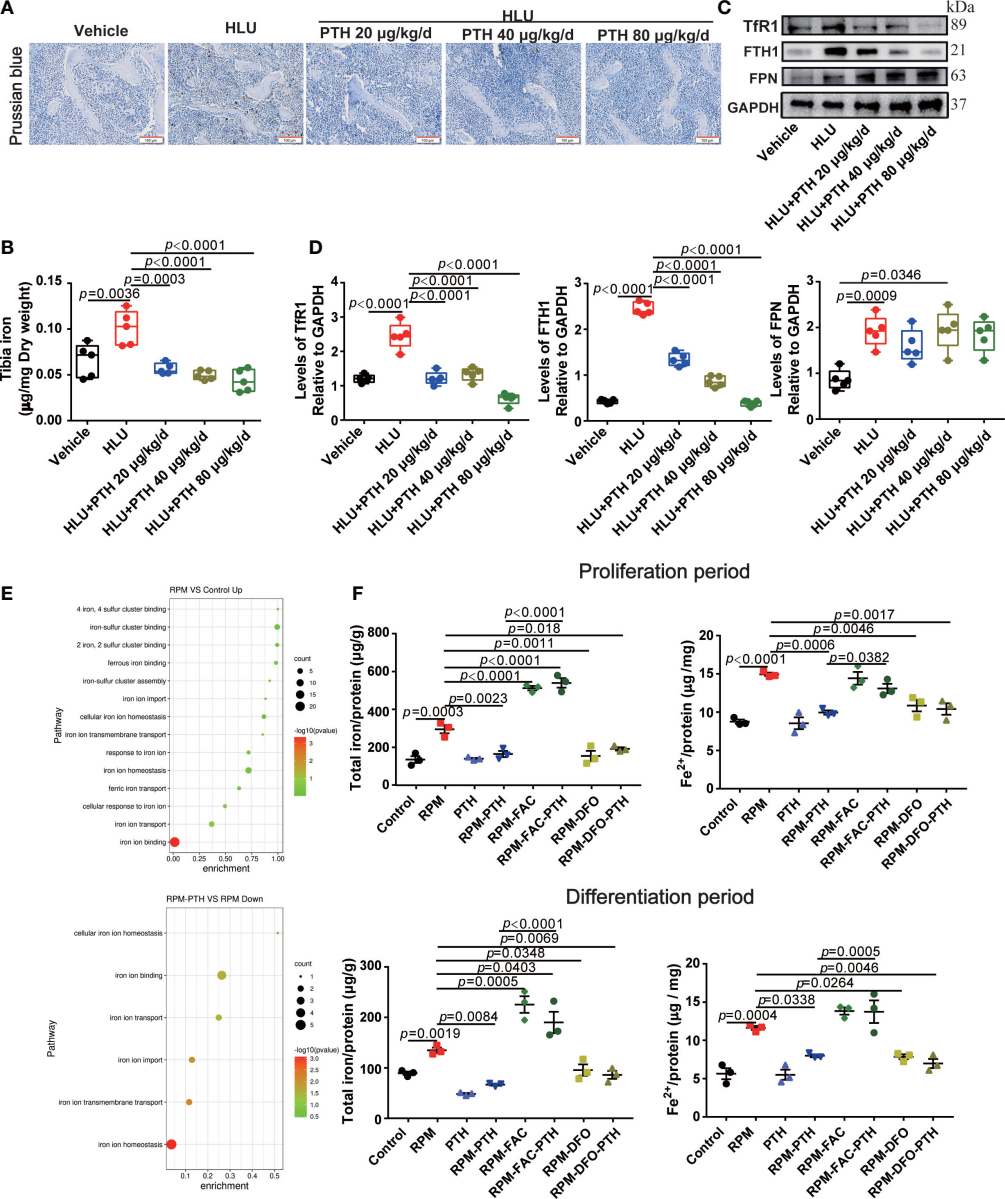
PTH 1-34 decreased iron content by regulating the expression of iron metabolism-related proteins both in the bone of HLU mice and osteoblasts under RPM conditions. **(A)** Iron staining of the femur in HLU mice by Prussian blue (scale bar = 100 μm). **(B)** The total iron content in the tibia of HLU mice was detected by AAS. **(C)** The expression of iron metabolism-related proteins in the femur of HLU mice was detected by western blot. **(D)** Quantification analysis of TfR1, FTH1 and FPN in bone. (n = 4). **(E)** GO enrichment classification map of differentially expressed genes in MC3T3-E1 cells under RPM conditions. **(F)** Total iron content was detected by AAS and ferrous ions detected by iron assay kit in MC3T3-E1 cells were pre-treated with or without FAC or DFO and treated with or without 100 nM PTH 1-34 under RPM conditions. (n = 3). Data is shown as the mean ± SEM.

Iron plays an important role in bone metabolism. Iron overload is a risk factor for bone loss (39). To reveal whether iron metabolism is involved in the PTH 1-34 promoted bone formation in HLU mice, we evaluated the changes in the iron content of bone tissue. These results showed that iron content was significantly increased in the bone tissue of HLU mice compared with that in vehicle groups, while iron content was decreased in a dose-dependent manner after HLU mice with PTH 1-34 treatment (**Figures 4A, B**). Next, we analyzed the expression levels of iron metabolism-related proteins in the tibia, and found that the expression levels of TfR1, FTH1 and FPN were increased in the HLU groups as compared to the vehicle groups, while PTH 1-34 effectively restricted the abnormal expressions of TfR1 and FTH1 in HLU mice (**Figures 4C, D**).

To explore whether iron metabolism in osteoblasts involved in the process of PTH 1-34 promotes bone formation in HLU mice, iron metabolism-related pathways were analyzed by transcriptome sequencing in MC3T3-E1 cells under RPM conditions with or without PTH 1-34 treatment for 48 h. The results showed that iron ion homeostasis, iron ion import, and iron ion transport were up-regulated in MC3T3-E1 cells under RPM conditions, while the iron ion import and iron ion transport were down-regulated by PTH 1-34 under RPM conditions (**Figure 4E**). Therefore, the iron metabolism was changed in the process of PTH 1-34 promoting bone formation under unloading conditions. To further verify the above results, the ferric ammonium citrate (FAC) and deferoxamine (DFO) were used to analyze the role of iron in process of PTH 1-34 promoting bone formation under RPM conditions. The results showed that the total iron and ferrous iron content in MC3T3-E1 cells were significantly increased under RPM conditions compared to the control group, while those were decreased under RPM conditions with PTH 1-34 treatment during the proliferation process or differentiation process (**Figure 4F**). what’s more interesting, the effect of PTH 1-34 reduction on total iron content and ferrous ions was blocked by the FAC (50 μM), while similar to DFO (10 μM) did in the proliferation or differentiation process of MC3T3-E1 cells under RPM conditions (**Figure 4F**). These results showed that PTH 1-34 may promote bone formation by regulating iron metabolism in bone tissue under unloading conditions.

### PTH 1-34 maintained the mitochondrial function and the REDOX balance by regulating iron metabolism in osteoblasts under unloading conditions

**Figure 5:**
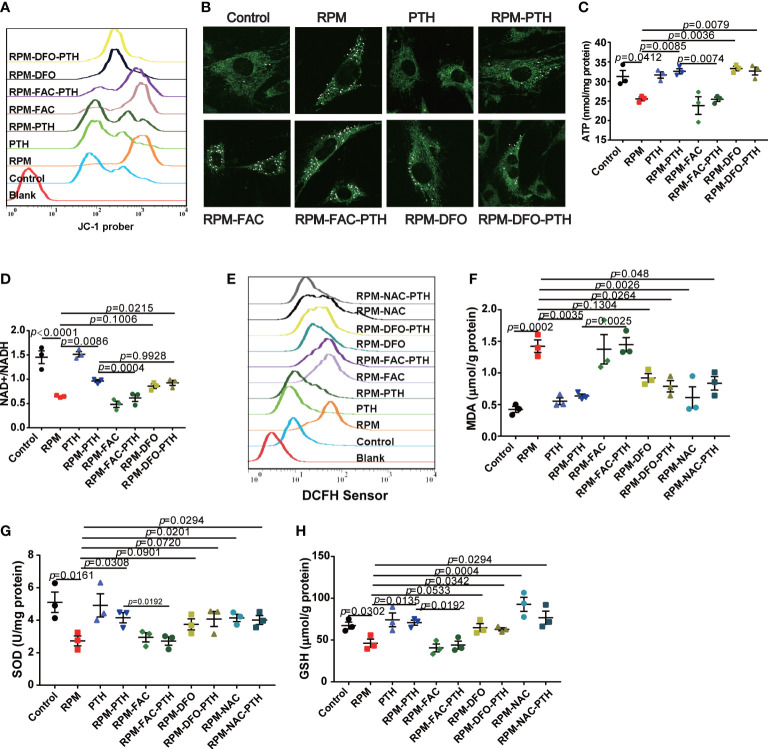
PTH 1-34 maintains mitochondrial function and the redox balance of osteoblasts under RPM conditions. **(A)** MMP in MC3T3-E1 osteoblast cells treated with or without PTH 1-34, FAC and DFO under RPM conditions for 24 h by JC-1. **(B)** Mitochondrial morphological changes in MC3T3-E1 osteoblast cells treated with or without PTH 1-34, FAC and DFO under RPM conditions for 24 h by MitoTracker Green. **(C**, **D)** ATP content and NAD+/NADH in MC3T3-E1 osteoblast cells treated with or without PTH 1-34, FAC and DFO under RPM conditions for 48 h. **(E)** ROS levels in MC3T3-E1 osteoblast cells treated with or without PTH 1-34, FAC, DFO and NAC under RPM conditions for 24 h by DCFH-DA sensor. **(F–H)** MDA, SOD and GSH content in MC3T3-E1 osteoblast cells treated with or without PTH 1-34, FAC, DFO and NAC under RPM conditions for 48 h. (n = 3). Data shown as the mean ± SEM.

Mitochondria has a key role in the REDOX balance, and the mitochondria function were regulated by many factors, including excess iron. Some studies showed that mitochondrion function was damaged in the microgravity environment. Then, we analyzed the mitochondria function by detecting intracellular MMP, mitochondria morphology, ATP content and NAD+/NADH ratio under RPM conditions. These results showed that the MMP significantly reduced under RPM conditions, while that was improved by PTH 1-34 under RPM conditions (**Figure 5A**), however, the effect of PTH 1-34 on MMP of MC 3T3-E1 cells was disturbed by FAC (50 μM), while that is similar to DFO (10 μM) (**Figure 5A**). Next, the result of mitochondria morphology showed that the mitochondria of MC3T3-E1 significantly rupture under RPM conditions, while that was inhibited by PTH 1-34 under RPM conditions, but this effect of PTH 1-34 on mitochondria disappeared when cells were pretreated with FAC (50 μM) (**Figure 5B**). ATP and NAD+/NADH are the markers to evaluate the mitochondria function. We analyzed the effect of PTH 1-34 on intracellular ATP production and NAD+/NADH ratio under RPM conditions. These results showed that the intracellular ATP production and NAD+/NADH ratio were decreased under RPM conditions, while PTH 1-34 significantly inhibited the reduction of them under RPM conditions (**Figures 5C, D**). Similarly, FAC (50 μM) disturbed these effects of PTH 1-34 promoting ATP generation and increasing NAD+/NADH ratio under RPM conditions (**Figures 5C, D**). These results indicated that PTH 1-34 protected mitochondria from damage by regulating iron metabolism under RPM conditions.

Mitochondria is the center of REDOX balance in cells, and excess ferrous iron catalyzes the ROS generation through the Fenton reaction to disturb the REDOX balance. Excess ROS induces the apoptosis of osteoblasts by inhibiting various signaling pathways, such as Wnt/β-catenin signaling and FoxOs-mediated transcription (40, 41). Following, we evaluated some markers of REDOX balance, including ROS levels, MDA content, SOD content and GSH content. These results showed that the ROS levels were significantly increased under RPM conditions compared with the control group, which were decreased by PTH 1-34 (**Figure 5E**). In addition, there were significantly increase of MDA (**Figure 5F**) and obvious decreases of SOD and GSH (**Figures 5G, H**) under RPM conditions compared to the control group, while PTH 1-34 prevented the change of them under RPM conditions. What’s more interesting, the ability of PTH 1-34 to maintain the REDOX balance disappeared when cells were pretreated FAC (50 μM), while it was not affected by DFO (10 μM) or NAC (1mM). These results further suggested that PTH 1-34 regulated the REDOX balance by regulating iron metabolism in MC3T3-E1 cells under RPM conditions.

### PTH 1-34 regulated iron metabolism by activating Nrf2 and hepcidin in the liver of HLU mice

**Figure 6:**
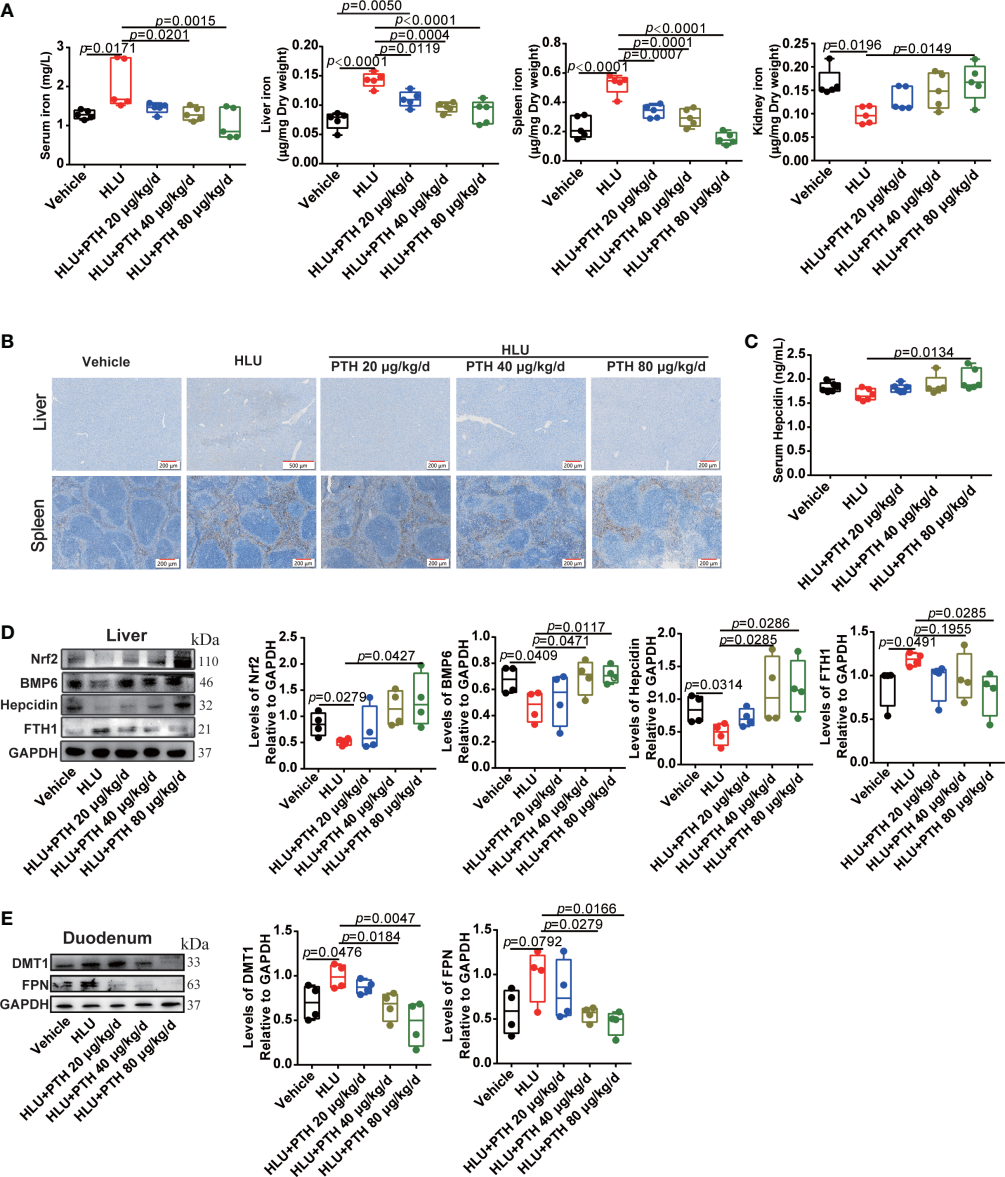
PTH 1-34 activated Nrf2 to enhance hepcidin expression in the liver for decreasing iron content in the bone of HLU mice. **(A)** The total iron content in the serum, liver, spleen and kidney of HLU mice was detected by AAS, (n = 5). **(B)** Iron staining of the liver and spleen in HLU mice by Prussian blue (scale bar = 100 μm). **(C)** The hepcidin content in the serum of HLU mice was detected by a mouse hepcidin ELISA kit, (n = 6). **(D)** The expression levels and quantitative analysis of iron metabolism-related proteins in the liver of HLU mice, (n = 4). **(E)** The expression levels and quantification analysis of iron metabolism-related proteins in the duodenum of HLU mice, (n = 4). Data is shown as the mean ± SEM.

The iron content of bone tissue is mainly derived from the input of systemic circulating iron. To evaluate whether PTH 1-34 decreased the iron content of bone tissue by inhibiting the increased systemic iron in HLU mice, iron content in serum, liver, spleen and kidney was detected. As shown in **Figure 6A**, iron content in serum, liver and spleen was significantly increased while decrease in kidney in the HLU group compared with that in the vehicle group, which was restored to near-normal level in a dose-independent manner by PTH 1-34 treatment in HLU mice. Liver and spleen are the major regulatory organ in systemic iron metabolism. Therefore, we further evaluated their iron deposition by Prussian blue staining. The results showed that iron was conspicuously deposited in the liver and spleen of the HLU group contrasted with that in the vehicle group, while PTH 1-34 reduced the iron deposition in liver and spleen of HLU mice (**Figure 6B**). Hepcidin, which was produced in the liver, is the master regulator of systemic iron homeostasis (42). Following, we analyzed the hepcidin content in the serum using a hepcidin ELISA kit. The result showed that the hepcidin was slight decrease in the HLU group compared to the vehicle group, while subcutaneous injection of PTH 1-34 (80 μg/kg/d) significantly enhanced the serum hepcidin content than that in the HLU group (**Figure 6C**). In addition, we analyzed the expressions of iron metabolism-related proteins in the liver and duodenum. These results showed that PTH 1-34 suppressed the expression of FTH1 in the liver, and the expression of FPN and DMT1 in the duodenum of HLU mice (**Figures 6D, E**). Nrf2 has been reported to play an important role in regulating iron metabolism. The absence of Nrf2 in the context of iron loading leads to both defective hepcidin regulation and tissue damage (43). Then, we analyzed the expression levels of NRF2 and BMP6 in the liver. The result showed that the expression of NRF2 and BMP6 in the liver of HLU group was significantly decreased compared with that of the vehicle group, while PTH 1-34 enhanced the expression of them, particularly 80 μg/kg/d (**Figure 6D**). These results suggested that PTH 1-34 regulated iron metabolism probably through activating the expression of Nrf2, which is further to promote hepcidin expression in the liver of HLU mice.

### The Nrf2 inhibitor partly blocked the effect of PTH 1-34 promoting bone formation by disturbing iron metabolism in HLU mice

**Figure 7:**
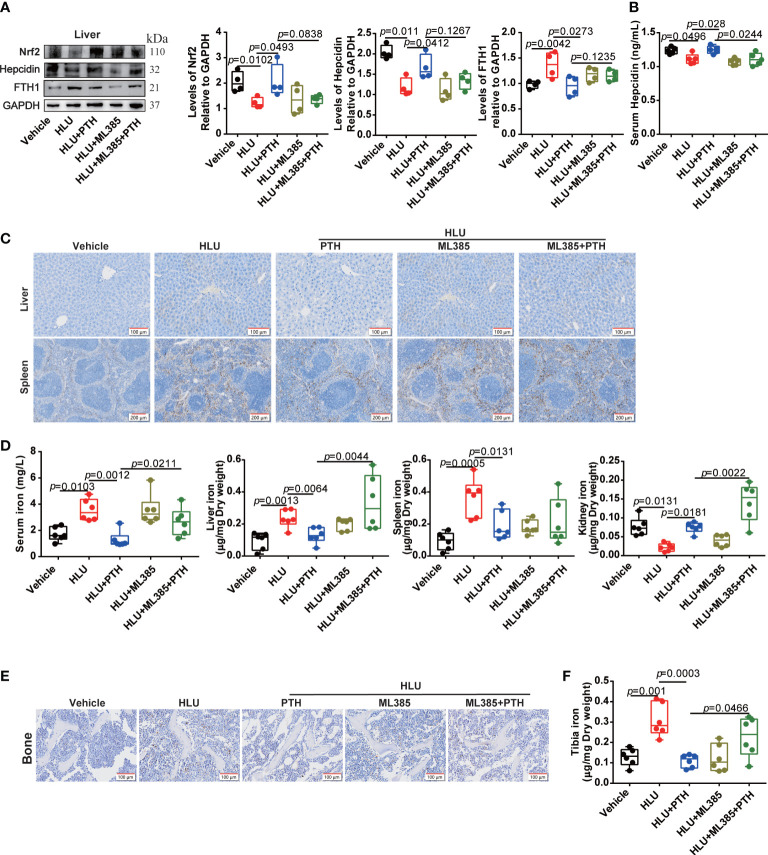
Nrf2 inhibitor blocked the effect of PTH 1-34 on iron metabolism regulation in HLU mice. **(A)** The expression levels and quantification analysis of iron metabolism-related proteins in the liver of HLU mice, (n = 4). **(B)** The hepcidin content was detected by mouse hepcidin ELISA kit in the serum of HLU mice, (n = 5). **(C)** Iron staining by Prussian blue in liver and spleen of HLU mice (scale bar = 100 μm). **(D)** The total iron content was detected by AAS in serum, liver, spleen and kidney of HLU mice, (n = 6). **(E)** Iron staining by Prussian blue in the bone of HLU mice (scale bar = 100 μm). **(F)** The total iron content was detected by AAS in the bone of HLU mice. (n = 6). Data is shown as the mean ± SEM.

Regulation of hepcidin by the activated Nrf2 may be involved in the promotion of bone formation by PTH-1-34 in HLU mice. Then, we used the Nrf2 inhibitor (ML385) to validate the effect of the Nrf2/hepcidin pathway in inhibiting bone loss by PTH 1-34 in HLU mice. ML385 was intraperitoneally injected at 30 mg/kg/7 d with or without PTH 1-34 (80 μg/kg/d, s.c.) in HLU mice. We found that ML385 inhibited the upregulation of Nrf2 by PTH 1-34 in the liver of HLU mice (**Figure 7A**). Following, we analyzed the effect of ML385 on the serum hepcidin and expression of hepcidin in liver, and found that ML385 attenuated the activation effect of PTH 1-34 on hepcidin in HLU mice (**Figures 7A, B**). In addition, the result of using ML385 combined with PTH 1-34 in HLU mice showed that there was significantly increased in the iron content in the liver, serum and kidney compared with that of the HLU +PTH 1-34 group (**Figures 7C, D**). For iron deposition and iron content in the bone of HLU mice, the results showed that ML385 attenuated the effect of PTH 1-34 on decreasing the iron deposition and iron content in the bone of HLU mice (**Figures 7E, F**).

**Figure 8:**
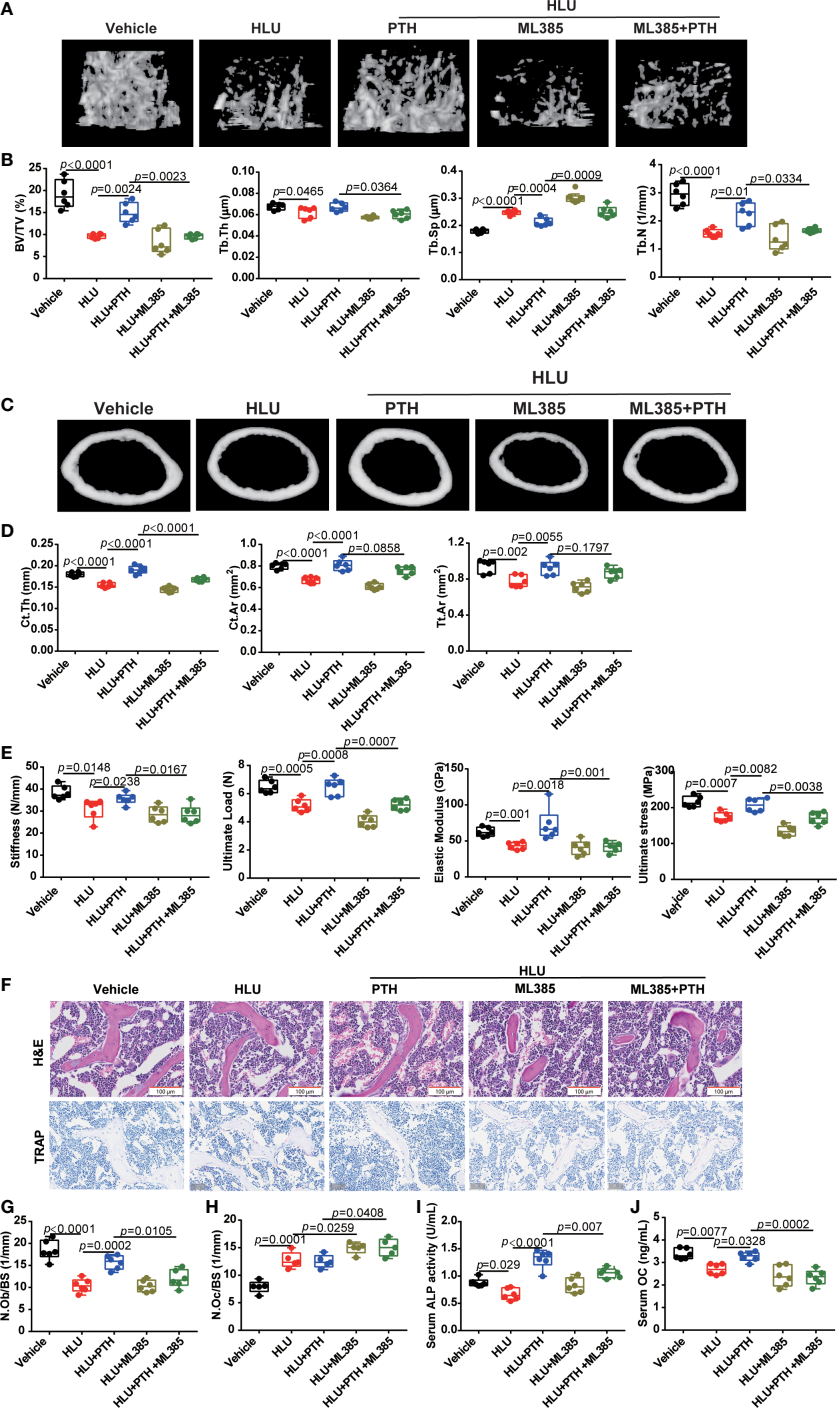
Nrf2 inhibitor blocked the effect of PTH 1-34 on promoting bone formation in HLU mice. **(A)** Three-dimensional images of trabecular architecture in the distal femur. **(B)** Structural parameters of trabecular bone, (n = 6). **(C)** Three-dimensional images of cortical architecture in the midshaft femur. **(D)** Structural parameters of cortical, (n = 6). **(E)** Mechanical properties of the tibia were detected through the three-point bending test, (n = 6). **(F)** H&E staining and TRAP staining of trabecular bone distal to the femur (scale bar = 100 μm). **(G)** The number of osteoblasts on trabecular surface was estimated by N.Ob/BS, (n = 6). **(H)** The number of osteoclasts on the trabecular surface was estimated by N.Oc/BS, (n = 5). **(I**, **J)** serum bone metabolism markers (OCN and ALP) were analyzed by ELISA kit, (n = 6). Data is shown as the mean ± SEM.

Then, we detected the role of ML385 in the effects of PTH 1-34 on bone formation in HLU mice. these results suggested that ML385 further worsened the femur microstructure of HLU mice with the decreased BV/TV, Tb.Th, and Tb.N, and increased Tb.Sp. More importantly, ML385 impaired the restorative role of PTH 1-34 in bone microstructure in HLU mice (**Figures 8A, B**). Application of ML385 reduced the protective ability of PTH 1-34 to the cortical bone by evaluating the Ct.Th, Ct.Ar and Tt.Ar in HLU mice, especially Ct.Th (**Figures 8C, D**). In addition, ML385 attenuated the protective effect of PTH 1-34 on mechanical properties by evaluating the stiffness, ultimate load, elastic modulus and ultimate stress in HLU mice (**Figure 8E**). To further verify the effect of ML385 on the bone anabolism of PTH 1-34, we measured the number of osteoblasts on the trabecular bone surface, serum ALP and OCN content. These results showed that ML385 inhibited the promoting effect of PTH 1-34 on bone formation in HLU mice by decreasing N. Ob/BS (**Figures 8F, G**), serum ALP content (**Figure 8I**), and serum OCN content (**Figure 8J**). Excess iron stimulates the osteoclasts differentiation. Then, the N.Oc/BS was analyzed by using TRAP staining. The result showed that the N.Oc/BS was further increased in the bone of HLU+ML385 groups mice compared with that of the HLU group, and increased in the bone of HLU+ML385+PTH groups mice compared with that of HLU+PTH groups (**Figures 8F, H**). Taken together, these results suggested that Nrf2/hepcidin was an important pathway in the promotion effect of bone formation by PTH-1-34 in HLU mice.

## Discussion

In the present study, we have shown that PTH 1-34 prevents bone loss by activating Nrf2 in the liver, which induced hepcidin expression to maintain systemic iron metabolism homeostasis in the HLU mice. For bone tissue, normal iron metabolism ensured bone formation and prevented the deterioration of bone microstructure in HLU mice. *In vitro* studies have shown that PTH 1-34 promoted osteoblast differentiation by regulating iron metabolism under RPM conditions. PTH 1-34 inhibited apoptosis of osteoblasts by regulating iron metabolism and maintaining the normal function of mitochondria under RPM conditions. Our study provides a new mechanism for understanding the bone anabolism of PTH 1-34 in osteoporosis induced by unloading through regulating iron metabolism balance. Nrf2 may be a potential therapeutic target for osteoporosis by PTH 1-34 treatment.

PTH is responsible for strictly regulating blood calcium levels. During bone unloading, a slight increase in serum calcium negatively regulates the PTH pathway. During space flight, the PTH content is decreased in the astronauts (44, 45). PTH also significantly decreased (about 29%) after 4 weeks of bed rest (46). In this study, we found that the serum PTH content was significantly decreased in mice by HLU treatment for 4 weeks. The pleiotropic effects of PTH are mediated by the PTH1 receptor (PTH1R) (47). PTH1R, a member of the G protein-coupled receptor (GPCR) family, can couple to either Gs or Gq depending on a tissue-type specificity (48). This receptor is almost ubiquitous, including bone and liver (16, 47). PTH1R is an important component of mechanical signal transduction for prolonging the lifespan of osteocytes (49). In this study, the expression of PTH1R in the liver and bone tissues of HLU mice was significantly reduced and was induced by the addition injection of PTH 1-34.

The drugs for treating osteoporosis in astronauts are mainly to inhibit bone resorption (such as Alendronate) (50). Inhibited bone formation is the primary factor in weightlessness-induced bone loss (51). As a clinically approved drug for promoting bone anabolism (52), the role of PTH in osteoporosis caused by weightlessness is controversial. Previous studies have shown that the bone anabolism of PTH is mechanically dependent, but the effect was alleviated in unloading-induced bone loss (53). Studies have also shown that PTH can inhibit bone loss caused by unloading in a dose-dependent manner (22, 23). In this study, high dose PTH 1-34 inhibited bone loss in HLU mice by promoting osteoblast differentiation and inhibiting osteoblast apoptosis. Our results provide new evidence for the therapeutic effect of PTH 1-34 on osteoporosis in HLU.

Iron overload and bone loss are intertwined. It is reported that excessive iron deteriorates the microstructure of bone tissue by disturbing the intracellular redox level (54, 55). In HLU mice, the duodenum absorbs more iron into the blood circulation, causing the formation of a large amount of free iron after the iron in blood circulation exceeds the ferritin binding capacity. The increase of intracellular unstable iron pools, which easily deposit free iron in various organs, especially in the liver (10, 56, 57). The relationship between PTH and its effect on iron metabolism has not been reported, but ferritin content and transferrin saturation are negatively correlated with PTH content as many studies reported (31). This may also explain why a weightless environment leads to increased iron content and decreased PTH content in the body. In this study, PTH 1-34 not only decreased the iron content of tibia bone in HLU mice but also maintain the REDOX balance by regulating iron metabolism in MC3T3-E1 osteoblasts. What’s more interesting, the effect of PTH 1-34 regulating iron metabolism was blocked by FAC, it was not affected by DFO. this main reason is possibility due to the similar target between PTH 1-34 and DFO in osteoblasts. Some studies showed that iron overload inhibited osteoblast differentiation by blocking Wnt/β-catenin pathway, while iron-chelating agents, including deferasirox (DFX), deferoxamine (DFO), Di-2-pyridylketone-4,4-dimethyl-3-thiosemicarbazone (Dp44mT) could promote cell proliferation by activating the Wnt/β-catenin pathway (58, 59). Wnt/β-catenin pathway also is the target to promote proliferation and differentiation of osteoblast by PTH 1-34. This indicated that the regulation of PTH on iron metabolism was involved in the bone anabolism of PTH 1-34 in HLU mice.

Excessive free iron can help to produce excess ROS through the Fenton reaction, causing the imbalance of redox homeostasis in the organs. Nrf2 is the main transcriptional regulator of cellular stress defense, coordinating the internal protective antioxidant response of cells (60, 61). During oxidative stress, Nrf2 transfers to the nucleus to form a dimer, which regulates the expression of hepcidin through the typical antioxidant response element in its promoter (43, 62, 63). In this study, we found that there was an increased content of iron in the liver, spleen, and serum of HLU mice, and more iron is absorbed into the system through the duodenum. The expression of hepcidin in the liver was reduced, leading to an imbalance of the hepcidin/FPN axis. More importantly, Nrf2, as a regulator of hepcidin, was also reduced in the liver of HLU mice. The iron content in tissue and system was further increased, and the expression of hepcidin was reduced by ML385 in HLU mice. Excessive iron in the circulatory system led to the imbalance of bone remodeling and exacerbated bone loss in HLU mice. The present study also confirmed that HLU mice had severe bone loss and decreased mechanical properties, accompanied by excessive iron accumulation. ML385 further deteriorated bone microstructure and mechanical properties, reduced bone formation ability, enhanced osteoclastogenesis, and resulted in iron accumulation in the system and organs.

Hepcidin is a hormone secreted by hepatocytes that plays a crucial role in regulating iron homeostasis (57, 64). Hepcidin regulates the systemic iron metabolism balance by blocking the iron release from macrophages, reducing iron release from hepatocytes and inhibiting iron absorption (57). BMPs, especially BMP6, are involved in hepcidin regulation (65, 66). Nrf2 is activated by iron and drives BMP6 expression in liver sinusoidal endothelial cells, which in turn increases hepcidin synthesis by neighboring hepatocytes (43). In the present study, the expression of Nrf2 was reduced in the liver tissues of HLU mice, which in turn led to a reduction in the expressions of BMP6 and hepcidin. ML385 decreased the expression of BMP6 and hepcidin in the liver and was accompanied by more iron absorption in HLU mice. The nuclear transport of Nrf2 can be activated in cells in response to external stimuli (oxidative stress) (61). In turn, Nrf2 alters iron homeostasis by increasing iron storage and reducing labile iron and can buffer labile iron by changing the flux of labile iron in and out of cells (62). Some studies have shown that Nrf2 is involved in the transcription of hepcidin (63) and PTH 1-34 can alleviate the production of excessive ROS in cells (29), but whether this process is achieved through the activation of nuclear transport of Nrf2 is not clear. In this study, we found that the expression of Nrf2 in the liver of HLU mice was significantly reduced, and this process was accompanied by an increase in iron content. we also found that the expression of Nrf2 in liver tissue was increased by PTH 1-34 in HLU mice. Then we injected the Nrf2 blocker into HLU mice and administrated with PTH 1-34 at the same time and found that Nrf2 blocker blocked the regulation level of PTH 1-34 to the iron content of liver tissue and system iron metabolism. More importantly, the Nrf2 blocker reduced the inhibitory effect of PTH 1-34 on osteoporosis in HLU mice. In summary, Nrf2 affects systemic iron metabolism and further regulates bone formation by regulating hepcidin expression in HLU mice, while the application of PTH 1-34 activates the nuclear transport of Nrf2 and regulates the level of iron metabolism in the system.

## Conclusion

This study proved that iron metabolism is involved in promoting bone formation by PTH 1-34 in HLU-induced bone loss, and also clarified the involved molecular mechanism. Combined with recent animal and human studies, our results further confirm the key link among iron metabolism, PTH 1-34 and bone loss in unloading conditions and possible molecular mechanisms. The current treatments for osteoporosis only focus on interventions in the process of bone resorption and bone formation. Our results indicate that we may miss the combined use of drugs on iron metabolism regulation during treatment. Therefore, our results not only confirm that iron metabolism is involved in the effect of PTH on promoting bone formation in HLU mice but also provide a new perspective for preventing bone loss of astronauts during space flight.

## Data availability statement

The datasets presented in this study can be found in online repositories. The data presented in the study are deposited in the SRA repository, accession number PRJNA924968.

## Ethics statement

The animal study was reviewed and approved by the Ethics Committee of Northwestern Polytechnical University.

## Author contributions

JC and PS designed the research. JC, WR, XC performed the experiments. JC and WR analyzed the data. JC wrote the original draft. FW and GZ reviewed and edited the draft. PS supervised the study. All authors contributed to the article and approved the submitted version.
